# What Can We Learn from Single Sarcomere and Myofibril Preparations?

**DOI:** 10.3389/fphys.2022.837611

**Published:** 2022-04-27

**Authors:** Walter Herzog

**Affiliations:** Faculty of Kinesiology, The University of Calgary, Calgary, AB, Canada

**Keywords:** titin, actin, myosin, mechanics of contraction, three-filament theory, residual force enhancement, passive force enhancement, serial sarcomeres

## Abstract

Sarcomeres are the smallest functional contractile unit of muscle, and myofibrils are striated muscle organelles that are comprised of sarcomeres that are strictly aligned in series. Furthermore, passive forces in sarcomeres and myofibrils are almost exclusively produced by the structural protein titin, and all contractile, regulatory, and structural proteins are in their natural configuration. For these mechanical and structural reasons single sarcomere and myofibril preparations are arguably the most powerful to answer questions on the mechanisms of striated muscle contraction. We developed and optimized single myofibril research over the past 20 years and were the first to mechanically isolate and test single sarcomeres. The results from this research led to the uncovering of the crucial role of titin in muscle contraction, first molecular explanations for the origins of the passive and the residual force enhancement properties of skeletal and cardiac muscles, the discovery of sarcomere length stability on the descending limb of the force-length relationship, and culminating in the formulation of the three-filament theory of muscle contraction that, aside from actin and myosin, proposes a crucial role of titin in active force production. Aside from all the advantages and possibilities that single sarcomere and myofibril preparations offer, there are also disadvantages. These include the fragility of the preparation, the time-consuming training to master these preparations, the limited spatial resolution for length and force measurements, and the unavailability of commercial systems for single sarcomere/myofibril research. Ignoring the mechanics that govern serially linked systems, not considering the spatial resolution and associated accuracies of myofibril systems, and neglecting the fragility of myofibril preparations, has led to erroneous interpretations of results and misleading conclusions. Here, we will attempt to describe the methods and possible applications of single sarcomere/myofibril research and discuss the advantages and disadvantages by focusing on specific applications. It is hoped that this discussion may contribute to identifying the enormous potential of single sarcomere/myofibril research in discovering the secrets of muscle contraction.

## Introduction

Muscle physiology is an exciting field that has been studied for thousands of years. Aristotle remarked on the muscular build of Olympic athletes more than 2000 years ago, contemplating their chances of winning, and Milo of Croton, a famous wrestler in ancient Greece, introduced increasing resistance training by lifting a young calf every day and as it grew heavier, requiring increasing strength to complete the task. In the late 15th and early 16th century, Leonardo da Vinci was famous for his immaculate drawings of the human body and its musculature, and in the 17th century, Giovanni Alfonso Borelli calculated the muscular forces required to perform working tasks.

However, muscle physiology as we know it today was formed by some of the greatest physiologists of the 20th century, Nobel Prize winners Otto Meyerhof, AV Hill, and AF Huxley. Their work contributed to our understanding of the muscle energetics, the force-length and force-velocity properties, and the molecular mechanisms underlying muscle contraction. Of particular interest to this treatise is the way we think about muscle contraction. In 1954, Hugh Huxley and Andrew Huxley (Huxley and Hanson, 1954; Huxley and Niedergerke, 1954) arrived independently at the idea that muscle contraction is caused by the sliding of two sets of filaments (actin and myosin) relative to each other, rather than the shortening of the myosin filament, as had been assumed prior to the 1950s. In 1957, Andrew Huxley then proposed a mechanism by which “side-pieces” (now called cross-bridges) from the myosin filament attach cyclically to the actin filament, pulling the actin past the myosin filaments with the energy provided by hydrolyzation of adenosine triphosphate (ATP) (Huxley, 1957). This theory, commonly known as the “cross-bridge theory”, is the way muscle contraction appears in textbooks of muscle physiology and is taught in courses of muscle mechanics to this day. Of course, some of the details of the 1957 theory have been revised, but the basic principles of two filaments sliding against each other, powered by cross-bridges and the energy derived from ATP, has remained untouched. For excellent, and highly personal accounts of the discovery of the cross-bridge theory, the associated technical developments, and the discovery of titin, please be referred to Maruyama (1995), Huxley (2004), and Dos Remedios and Gilmour (2017).

**FIGURE 1:**
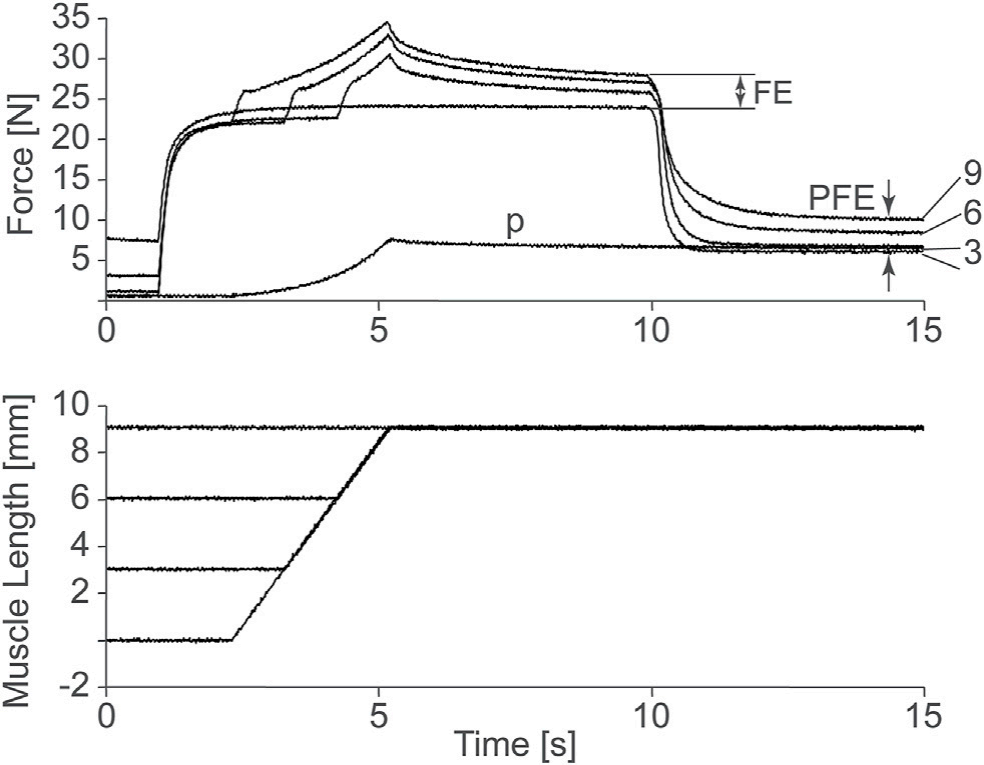
Force as a function of time of a cat soleus muscle stretched 3, 6, and 9 mm, or about 3, 6, and 9% of its total muscle tendon until length. Observe the increase in steady-state isometric force following the active stretches compared to the corresponding purely isometric reference contraction at the final length. This so-called residual force enhancement (FE) increases with increasing stretch magnitude. Also note the increase in passive force following deactivation of the actively stretched muscle compared to the passive force obtained in the passively (P) stretched muscle. This so-called passive force enhancement (PFE) also increases with increasing active stretch magnitudes. Herzog and Leonard, 2002, permission pending.

At this point, it is important to note that the cross-bridge theory has been used successfully to predict muscle force, stiffness, and energetics for shortening (concentric) and constant length (isometric) muscle contractions but has failed to predict these mechanical parameters with equal success for lengthening (eccentric) muscle action (Huxley, 1957). Huxley (1957) was aware of the shortcomings of his initial theory, and remarked greatly on it, and proposed solutions for how the cross-bridge theory may remain untouched and still predict the mechanics of actively lengthening muscles (e.g., Huxley, 1980). In his beautiful book “Reflections on Muscle”, Huxley remarked that “special features” may have evolved in muscle to account for the mechanics of eccentric muscle action, and that possibly the development of sarcomere length non-uniformities during active muscle lengthening might be responsible for the unexplained properties observed in lengthening muscle (Huxley, 1980). Among the many unexplained properties of lengthening muscle, two stand out: 1) the long-known increase in steady-state force following active muscle stretching, now referred to as “residual force enhancement” (Abbott and Aubert, 1952; Edman et al., 1982), and 2) the more recent discovery of an increase in passive force during and following active muscle stretching, discovered by us in 2002 in experiments on cat soleus muscle, and now referred to as “passive force enhancement” (Herzog and Leonard, 2002). Passive force enhancement is the increase in (passive) force after a muscle has been actively stretched and then deactivated (Figure 1). It is most prominent and best observed in muscle preparations that are stretched to lengths where passive force occurs naturally. Passive force enhancement is long-lasting (minutes), increases with increasing active stretch amplitude, and is seen across all structural levels, including single sarcomeres, and thus is speculated to be associated with titin.

The discovery that the passive force, force not created by the contractile filaments actin and myosin, is increased in active compared to passive muscle, changed the way many scientists thought about muscle contraction and brought up two fundamental questions: 1) what passive element(s) in muscle causes this increase in passive force when a muscle is activated and stretched, and 2) what are the molecular mechanisms underlying this increase in passive force? These two questions, we felt, could not be answered using traditional methodologies, but required the complete control of individual sarcomeres. Research on a higher structural level than the sarcomere, single fibres for example, are deemed not useful in this context, as the crucial variables of individual sarcomere forces and individual sarcomere lengths, cannot be determined uniquely, as there is no possibility to measure or theoretically determine sarcomere forces in this highly mechanically redundant system, and a technique to measure the thousands of individual sarcomere lengths simultaneously in a single muscle fibre does not exist. Research on a lower structural level, for example using isolated cross-bridges and actin filaments, as introduced in laser trapping experiments by Finer et al. (1994), were also deemed less meaningful than myofibril preparations, as the intricate structural integrity of the sarcomere, that we believe plays a crucial role in the molecular details of muscle contraction, is lost.

For these reasons, we started to develop techniques to work with single myofibril and single, mechanically isolated sarcomere preparations. The topic of this special issue is on methods used in striated muscle mechanics and physiology, and here, we would like to describe the methods for single sarcomere and myofibril preparations. However, the technical details of these preparations can be obtained in any of the many manuscripts we (and others) have published on the topic, and thus, they will just be described briefly. More importantly, when working with single myofibrils and sarcomeres, information can be obtained that cannot be gathered from other muscle preparations. We will focus on these advantages of myofibrils over other structural preparations but will also point out the limitations of working with myofibrils and the errors that have been made by not recognizing and acknowledging these limitations.

## Methods

### Single Myofibril Preparation for Mechanical Testing

Typically, we work with rabbit psoas myofibrils, but myofibrils from other muscles can be prepared in a similar manner. We isolate strips of rabbit psoas muscle from euthanized animals using forceps and tie them to wooden sticks to preserve the *in-situ* sarcomere length and prevent shrinking. These strips are then placed in a rigor-glycerol solution with protease inhibitors (Complete, Roche Diagnostics, Montreal, QB, Canada) and are then stored at −20°C for about 12 ± 2 days. On the day of experimentation, the muscle strips are placed in a +4°C rigor solution, homogenized, and placed in the experimental chamber that is typically held at room temperature of 20°C.

**FIGURE 2:**
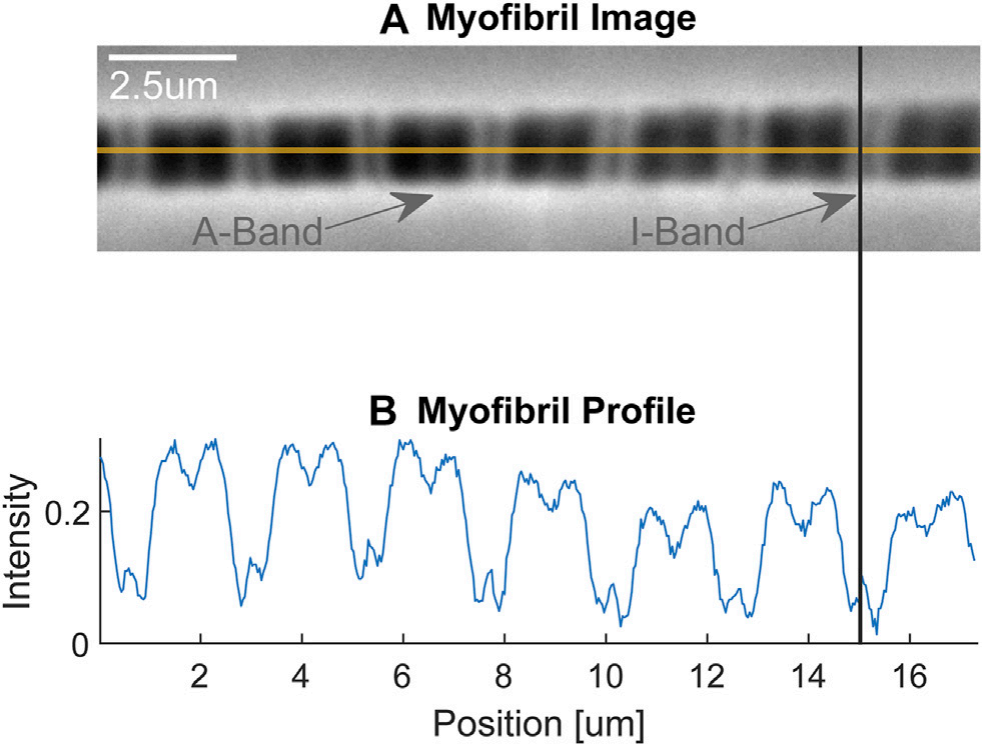
**(A)** Single myofibril with dark-light striation pattern. The dark areas represent the A-bands containing the thick (myosin) filaments, while the light areas represent the I-bands containing the thin (actin) filaments. The faint dark line in the approximate centre of the I-bands is the Z-line delineating the end of the sarcomeres (indicated also with the vertical line connecting figures **(A,B)**. The myofibril is attached on its left-hand end to a rigid glass pipette, which in turn is attached to a piezo-tube motor that allows for nano-metre step size control (not shown) and is attached on its right-hand end to a silicon nitride nano-lever for force measurements (not shown). **(B)** Light intensity pattern of the myofibril shown in **(A)**. The dark, A-bands are given high intensity values, while the light, I-bands, are given small intensity values. Republished with permission of Royal Society, from Dynamics of individual sarcomeres during and after stretch in activated single myofibrils. Rassier DE, Herzog W, Pollack GH. Proc. R Soc Lond Volume 270, *p*. 1735–1740. 2003; permission conveyed through Copyright Clearance Center Inc.

The mechanical testing is done by attaching the isolated myofibrils to a force transducer at one end and a glass pipette attached to a motor at the other end (Figure 2). Attachment to the force levers is either done by wrapping the myofibril around the force lever, or by gluing the myofibril to the force lever. Attachment to the rigid glass pipette is done by attaching a small amount of glue to the end of the pipette and then piercing the pipette into the z-line region of the end sarcomere of a myofibril. Experiments are performed on the stage of an inverted light microscope. We typically use a ×100 oil immersion objective (NA 1.3) and have the possibility to add a ×2.5 Optovar configuration for a total magnification of ×250. Depending on the question, we use myofibrils containing one (single sarcomere preparation) and up to about 20 sarcomeres. Individual sarcomere lengths are measured continuously using a linear photo diode array camera. Frequently, we define sarcomere lengths from the centroid of the A-band of one sarcomere to the centroid of the A-band of the adjacent sarcomere, but in a good preparation, where the Z-lines can be identified clearly, we measure sarcomere lengths in the traditional way from centroid to centroid of adjacent Z-lines. We use a glass pipette attached to a custom-built piezo-tube motor to perform any desired length change of a myofibril for mechanical testing. Passive and active forces in the myofibrils are measured using custom-built nanofabricated silicon nitride cantilevers with stiffness ranging from 20–200 pN/nm depending on the forces expected in active or passive experiments at different sarcomere lengths, and for shortening, isometric and lengthening contractions, all of which affect the force capacity of the myofibril. Knowing the stiffness of the nano-levers, force can be calculated by the displacement of the lever from its resting position. Typically, forces are normalized relative to the cross-sectional area for comparison between myofibrils of different size.

The great advantage of a single myofibril preparation is that all sarcomeres are strictly arranged in series. This in series alignment ensures that each sarcomere and half-sarcomere transmits the same force and that this force can be measured at the end of the myofibril (neglecting any inertial effects and mass movements, which can safely be done in myofibril preparations). More details on the properties of in series mechanical systems, how they behave, and how they can be represented mathematically, may be obtained in Epstein et al. (2006). Since a single myofibril also allows for the determination of each sarcomere and half-sarcomere length, the instantaneous force, sarcomere length, and sarcomere rate of change in length (speed of contraction) are known at any instant in time, allowing for a complete description of the mechanics of all sarcomeres. However, the concept of a system of elements that are mechanically “in series” must be treated properly, otherwise it may lead to incorrect interpretations of results and misleading conclusions, as has happened in some recent works (Mendoza and Rassier, 2020).

### Single Sarcomere Isolation for Mechanical Testing

For mechanical isolation and testing of a single sarcomere, a short myofibril consisting of 5–10 sarcomeres is attached at both ends as described above using micro-manipulators that allow for movements in the sub-nano-metre range. Once attached in this manner, the sarcomere closest to the force lever is isolated by using a third micromanipulator with a stiff glass pipette and attaching it to the Z-line of that sarcomere that is away from the force lever. This third micromanipulator is then attached to the piezo-tube motor that is used for imposing length changes to the sarcomere (Leonard et al., 2010). It is a powerful but technically difficult preparation to set up properly and obtain meaningful results (Herzog et al., 2016; Schmidt et al., 2021).

One of the great advantages of the single sarcomere preparation is that the complex interaction of parallel and serially arranged sarcomeres, as they occur in single fibre and single myofibril preparations respectively, is eliminated. Therefore, the mechanical properties of this basic contractile unit of muscle can be measured without interference caused by sarcomere length non-uniformities, force transmission across the Z-lines, uneven activation of sarcomeres in a myofibril, single fibre or muscle preparation, and without passive force contributions by structural proteins and the collagen matrix network except for the forces produced by the molecular spring titin (Bartoo et al., 1997) (Linke et al., 1994) (Colomo et al., 1997) (Linke, 2018).

## Applications

There are dozens of applications that have shed light into the mechanisms of muscle contraction in general, and the role of titin specifically, using single myofibril and single sarcomere preparations. Here, we would like to briefly mention three such applications that have changed the way we think about muscle contraction today as compared to just a decade ago:

1) The discovery of titin as a spring of variable stiffness.
2) The discovery of residual force enhancement in a single sarcomere.
3) The discovery of activation-dependent unfolding of titin segments.

### Titin as a Spring of Variable Stiffness

**FIGURE 3:**
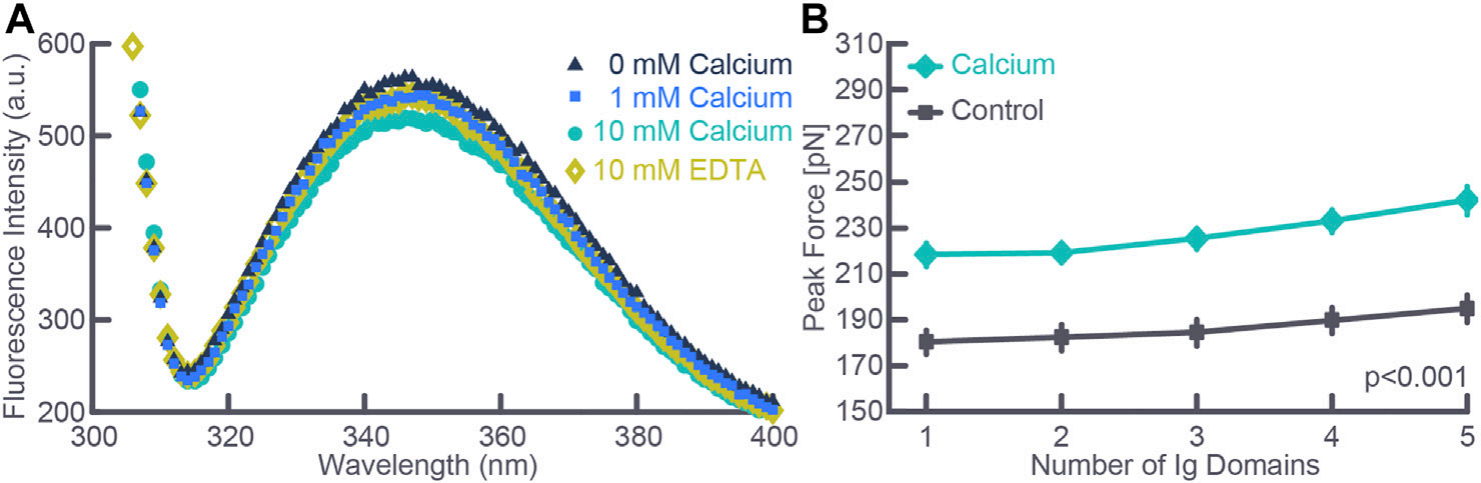
**(A)** Fluorescence intensity measured using spectroscopy as a function of calcium concentration for an exemplar test. Note the depression of the peak intensity fluorescence with increasing calcium concentration and the reversal of the peak fluorescence intensity when removing free calcium using EDTA. **(B)** Force required to cause unfolding of five immunoglobulin domains in the absence (Control) and the presence (Calcium) of physiologically relevant calcium concentration. Note that the force required for immunoglobulin unfolding increased by about 20% in the presence of calcium. Note also, the slight increase in force required with each unfolding event despite identical immunoglobulin domains which is explained by the stochastic nature of the unfolding events. The total number of observations for each data point was greater than 300 in all cases, and data points where the standard deviation bars cannot be seen indicates that the standard deviations were smaller than the size of the data point. Republished with permission of American Physiological Society, from Passive force enhancement in striated muscle. Herzog W. Journal of Applied Physiology 126 (6): 1782–1789. 2019; permission conveyed through Copyright Clearance Center Inc.

With the discovery of the passive force enhancement property in skeletal muscle, we proposed that titin might cause passive—and at least some of the residual—force enhancement in skeletal muscle (Herzog and Leonard, 2002). This proposal was supported in elegant experiments by Labeit et al. (2003), who studied recombinant PEVK segments of titin containing two highly conserved elements. Using single molecule experiments they showed that calcium-induced conformational changes altered the stiffness in these conserved PEVK fragments. Further experiments by Labeit et al. (2003) using skinned single fibers from the mouse soleus showed that titin-based force was dependent on activation. Experiments on myofibril preparations arrived at the same conclusion when single myofibrils from rabbit psoas were stretched in baths containing different concentrations of calcium. The advantage of the single myofibril over the single fibre preparation was that titin-based forces could be directly quantified by inhibiting cross-bridge based forces either chemically (Leonard and Herzog, 2010) or by selective deletion of regulatory proteins (Joumaa et al., 2008) and knowing that titin was the only structural element in sarcomeres contributing to the passive force (Bartoo et al., 1997). Further experiments on recombinant immunoglobulin domains of cardiac titin also revealed that calcium binds to these segments in a dose-dependent manner, that calcium binding is reversed when calcium is removed, and that unfolding the immunoglobulin domains in the presence of calcium requires about 20% more force than the corresponding unfolding forces in the absence of calcium (Figure 3) (Duvall et al., 2013). The conclusion from these studies was that titin is a calcium dependent molecular spring; that is, the stiffness of titin changes depending on the active state of a muscle.

### Residual Force Enhancement in a Single Sarcomere

For approximately the past 70 years, it has been believed that sarcomere lengths and sarcomere forces are unstable once sarcomeres reach lengths greater than their optimal lengths (Hill, 1953); that is, when sarcomeres operate on the descending limb of the force-length relationship where actin-myosin filament overlap decreases with increasing length, thereby reducing the number of possible cross-bridge attachments between actin and myosin, thus decreasing the steady-state, isometric force. This notion of instability led to the idea that muscles stretched on the descending limb of the force-length relationship exhibit residual force enhancement because of the development of sarcomere length non-uniformities. A basic premise of this theory is that purely isometric contractions are associated with essentially uniform sarcomere lengths even on the descending limb of the force-length relationship, while stretching an active muscle on the descending limb causes rapid elongation of “weak” sarcomeres, causing great non-uniformities in sarcomere length that then give rise to the residual force enhancement (e.g., Morgan, 1994). This theory has support to this day, including from experiments using single myofibrils (e.g., Rassier and Pavlov, 2012), even though it has been shown unequivocally that vertebrate muscles are inherently and naturally comprised of sarcomeres of highly non-uniform lengths even in the passive state and during isometric contractions (Huxley and Peachey, 1961; Llewellyn et al., 2008; Johnston et al., 2016; Moo et al., 2016).

**FIGURE 4:**
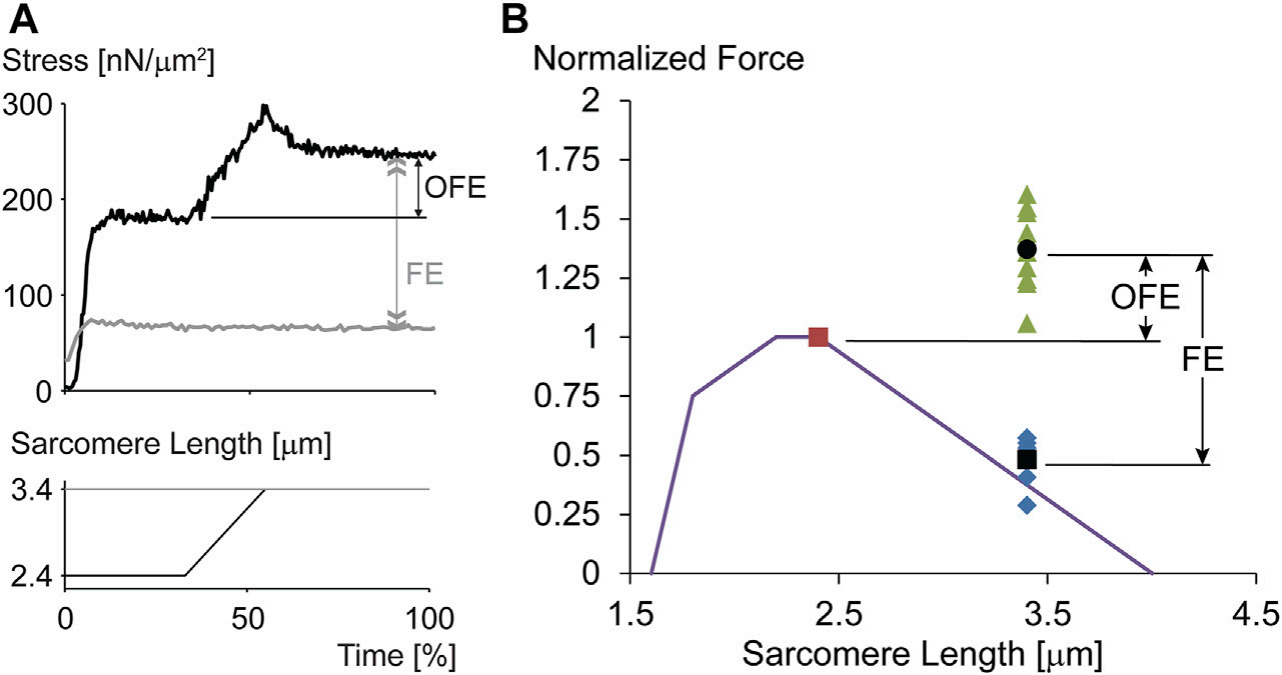
**(A)** Force-time and sarcomere length-time trace for an exemplar experiment performed on a single, mechanically isolated sarcomere. The isometric reference contraction at a sarcomere length of 3.4 µm is shown in gray, and the corresponding force enhancement test (stretch from a sarcomere length of 2.4–3.4 µm) is shown in black. Note that the initial isometric contraction for the force enhancement test (black trace) was performed at optimal sarcomere length for the rabbit psoas sarcomere used here. Observe the dramatic force enhancement, FE (greater than 200% in this example), and the substantial force above the maximal isometric force at optimal length (OFE). **(B)** Theoretical sarcomere force-length relationship for rabbit skeletal muscle and isometric reference force obtained at optimal sarcomere length (red dot, normalized to 1.0), the corresponding isometric reference forces obtained at a sarcomere length of 3.4 µm, and the corresponding isometric, steady state forces obtained after stretching single sarcomeres from 2.4 to 3.4 µm while activated. The average force enhancement (FE; average values are indicated by the black dots) was 185% and the average enhanced forces exceeded the isometric optimal reference forces (OFE) by 38%. In summary, single mechanically isolated sarcomeres show great residual force enhancement and exceeded the isometric references forces at optimal sarcomere length in all cases when stretched from optimal (2.4 µm) to the final (3.4 µm) sarcomere length. Republished with permission of American Physiological Society, from Residual force enhancement following eccentric contractions: a new mechanism involving titin. Herzog W., Schappacher Tilp G, DuVall M, Leonard TR, Herzog J. Physiology 31: 300–312. 2016; permission conveyed through Copyright Clearance Center Inc.

The sarcomere length non-uniformity theory can be used to make detailed and testable hypotheses about the properties of residual force enhancement. The most prominent of these hypotheses are that residual force enhancement cannot occur on the ascending limb of the force-length relationship (as there is no sarcomere length instability on that part of the relationship), residual force enhancement cannot exceed the plateau forces of the isometric force-length relationship (as the enhanced forces are limited to what the active sarcomeres can produce at optimal actin-myosin filament overlap), and residual force enhancement is associated with sarcomere length non-uniformities that are greater than the essentially uniform sarcomere lengths assumed for isometric contractions. All these predictions of the sarcomere length non-uniformity theory have been rejected in multiple experiments (e.g., Rassier et al., 2003; Peterson et al., 2004; Rassier, 2012; Johnston et al., 2019). Nevertheless, the ultimate proof that residual force enhancement does not depend on the development of non-uniformities in sarcomere lengths but is an inherent property of muscle, did not exist until residual force enhancement was shown to occur in single, mechanically isolated sarcomeres. For this reason, we developed the single sarcomere preparation that allowed mechanical experiments and the determination of residual force enhancement in this basic contractile unit of muscle. Not only was it shown that residual force enhancement existed in each of the single sarcomeres tested (Figure 4), but the force enhancement following a stretch from 2.4 to 3.4 µm averaged an impressive 185% and the average enhanced force exceeded the maximal isometric force at the plateau of the force-length relationship by 38% (Leonard et al., 2010). Since force enhancement needs to exist in both half-sarcomeres, the argument that a non-uniformity in the two half-sarcomeres could explain the results is not valid. These experiments demonstrated that the residual force enhancement property is contained within a sarcomere and does not require the development of non-uniform sarcomeres. However, it does not exclude the possibility that sarcomere length non-uniformities might contribute to the residual force enhancement on top of the force enhancement obtained due to the sarcomere internal mechanics. In fact, in some experiments using single myofibrils and sarcomeres it was concluded that residual force enhancement has a “component associated with half-sarcomere dynamics, which significantly increases the level of force enhancement after stretch.” (e.g., Rassier and Pavlov, 2012). However, these results (Figure 8A in Rassier and Pavlov, 2012) must be considered with caution as the half-sarcomere length non-uniformities measured were given in ∼0.4 nm increments with a maximal value of 12 nm. Such accuracy cannot be achieved with the light microscopy used in that study and comprises random noise. For sub-nm accuracy of contractile specimens, approaches such as low angle x-ray diffraction are required (Ma and Irving, 2019). Sarcomere and half-sarcomere length changes with light microscopy need to exceed about 100 nm for reliable detection (Schmidt et al., 2021).

In summary, experiments on single, mechanically isolated sarcomeres demonstrate that residual force enhancement is a property inherent to a single sarcomere. Non-uniformity of sarcomeres or half-sarcomeres has not been demonstrated to contribute directly to residual force enhancement.

### Activation-Dependent Unfolding of Titin Segments

**FIGURE 5:**
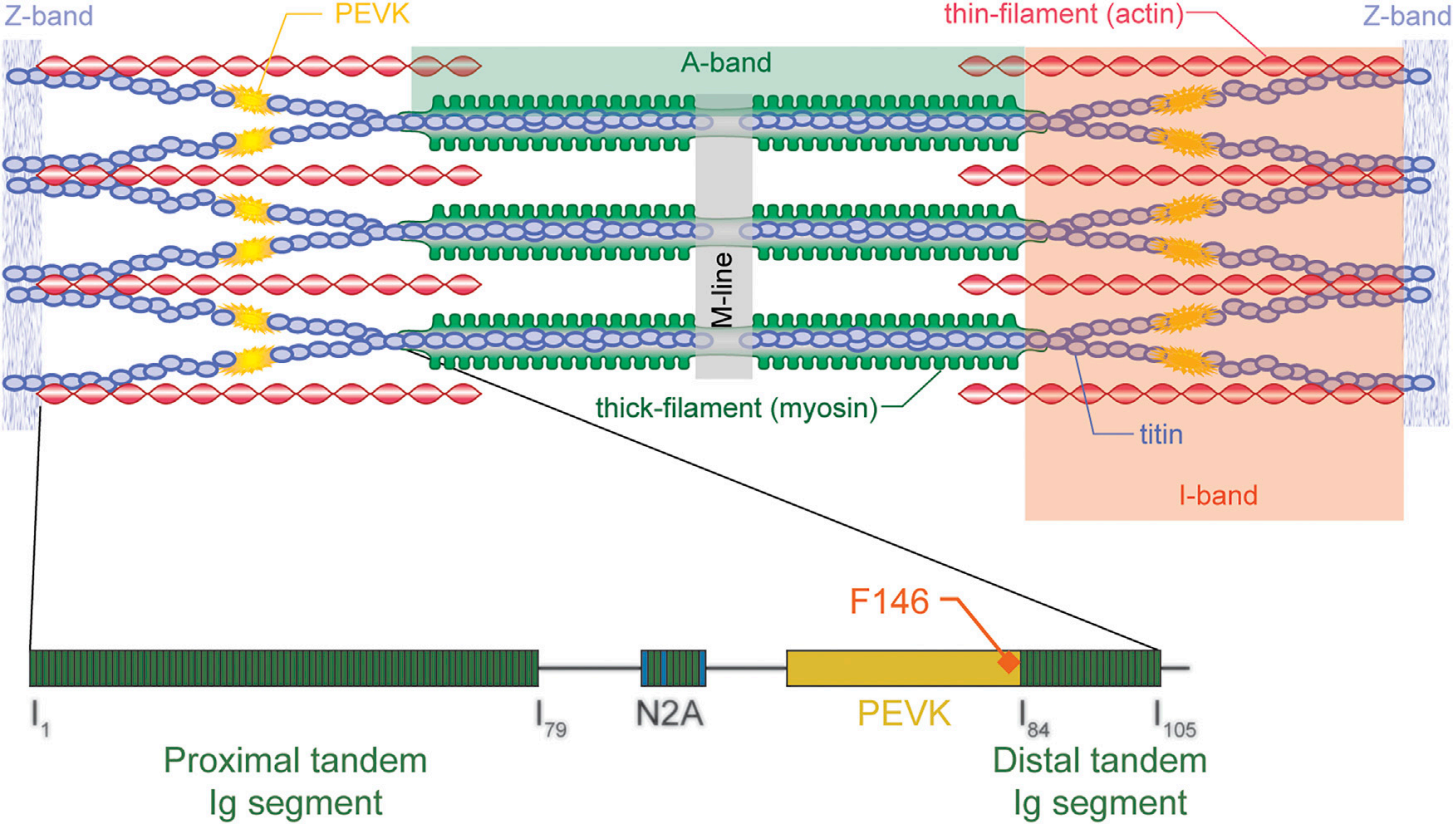
Schematic illustration of a sarcomere (top) with actin, myosin, and titin (and titin’s molecular structure - bottom) indicated. Titin inserts into the M-line in the middle of the sarcomere and the Z-line at the end of the sarcomere, thus spanning the entire half-sarcomere. Titin is “rigidly” bound to the myosin filament in the A-band region but runs freely across most of the I-band region, except at the very proximal end where it merges with actin just prior to inserting into the Z-line. In the I-band region, titin elongates, thus producing passive force when a sarcomere is stretched. For physiologically relevant elongations of skeletal muscles, titin’s immunoglobulin become aligned and the PEVK segment elongates. Only towards the end of physiologically relevant elongations, or when the muscle is active (rather than passive) is it thought that immunoglobulin (Ig) domains unfold. Shown is also the F146 antibody (for further details see text below) which attaches to the distal end of the PEVK domain of titin. Republished with permission of John Wiley & Sons, Inc., from Structure-function relations of the giant elastic protein titin in striated and smooth muscle cells. Granzier H, Labeit S. Muscle & Nerve Volume 36, *p*. 740–755. 2007; permission conveyed through Copyright Clearance Center Inc.

Titin is the third most abundant protein in skeletal muscle. It extends from the M-band across the half sarcomere and inserts into the Z-line bound to actin (Figure 5). Titin acts like a molecular spring in the I-band region and its segments unfold sequentially when a muscle/sarcomere is stretched, and then refold upon muscle/sarcomere shortening (Linke et al., 1999; Granzier and Labeit, 2002; Labeit et al., 2003). Titin steadies the myosin filament in the centre of the sarcomere, provides passive force, and is implicated in force signaling (Horowits et al., 1986; Horowits and Podolsky, 1987; Horowits, 1992; Bartoo et al., 1997; Herzog, 2018; Schappacher-Tilp, 2018). In 2002, when discovering that passive forces were greater in actively compared to passively stretched muscles, we proposed that I-band titin is an activatable spring that can change its stiffness, and thus its force contribution when a muscle is stretched. Even though the idea that titin is an adjustable spring had been formulated before (e.g., Helmes et al., 1999; Freiburg et al., 2000; Granzier and Labeit, 2002), these ideas were typically in the context of long-term adaptations, expressions of different isoforms, and changes caused by post-translational modifications. Our suggestion was that titin could adjust its spring stiffness “instantaneously” by changing its inherent spring length when a muscle was activated and produced actin-myosin-based active force. Two possibilities were suggested for the change in titin stiffness: 1) calcium binding to specialized segments of titin, thereby increasing the force required for unfolding of these segments, and 2) titin shortening its free spring length by attaching its proximal segment to actin (e.g., Leonard and Herzog, 2010). The proximal segment was proposed since titin is known to be permanently bound to actin at its proximal end, approximately 80–100 nm from inserting into the Z-line (Trombitas and Granzier, 1997).

**FIGURE 6:**
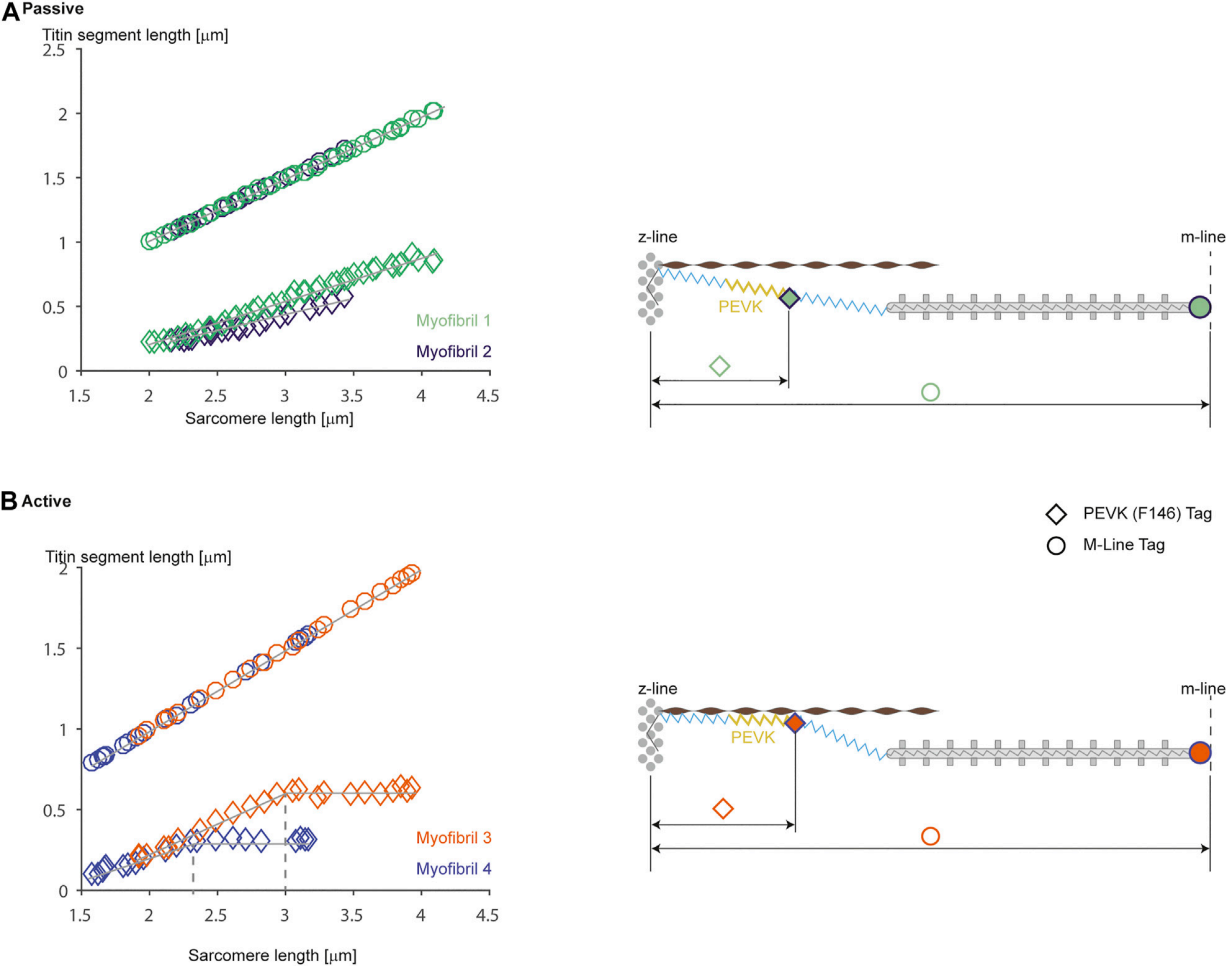
Passive **(A)** and active **(B)** stretching of proximal titin segments labeled using an antibody [F146 that binds to the PEVK region (diamond symbols)] region that allows for measurements of proximal and distal titin segment elongations during passive and active stretching of single rabbit psoas sarcomeres. Figures on the left show elongation of the half-sarcomere [top traces (circular symbols) using an M-line label] and elongations of the proximal titin segment (bottom traces: from the Z-line to the F146 label) for two representative sarcomeres from two different myofibrils. Note in **(A)** (passive stretching) that the two proximal titin segments elongate continuously with half-sarcomere elongations, reaching final lengths of approximately 0.95 μm (at a sarcomere length of 4.0 μm) and about 0.6 μm (at a sarcomere length of about 3.5 μm). In contrast, when the myofibrils are stretched while activated, the proximal segments elongate similarly to the elongations observed in the passive condition, but then stop elongating and remain substantially shorter than in the passive case (i.e., with a length of about 0.6 and 0.35 μm, respectively). The panels on the right illustrate schematically what we believe might be happening. In the passive stretch **(A)**, the proximal and distal titin segments elongate in accordance with their stiffness properties. In the active stretch **(B)**, titin is thought to attach to actin at some point during the stretch, thereby shortening titin’s free spring length, increasing its stiffness, eliminating elongation of the proximal titin segment, and increasing titin-based force. Republished with permission of American Physiological Society, from Residual force enhancement following eccentric contractions: a new mechanism involving titin. Herzog W., Schappacher Tilp G, DuVall M, Leonard TR, Herzog J. Physiology 31: 300–312. 2016; permission conveyed through Copyright Clearance Center Inc.

In order to determine the elastic properties of titin in passive and active muscle, Horowits et al. (1989) used titin antibody labeling to measure the extension of proximal and distal titin segments in skinned rabbit psoas fibers using immunoelectron microscopy. They concluded that titin remained rigidly attached to the myosin filament and that the I-band elastic properties of titin were unaffected by activation. However, following antibodies in whole fibre preparations is difficult due to the large number of labels and the distortions of parallel aligned myofibrils within a fibre. Using titin specific antibodies in rabbit psoas myofibrils, we observed distinct differences in the unfolding characteristics of titin segments in actively and passively stretched myofibrils. In passively stretched myofibrils, titin elongated according to its known elastic properties in its entire I-band length (Figure 6). In actively stretched myofibrils, the proximal segments stopped elongating after a small stretch, suggesting that the proximal segment had become infinitely stiff. The interpretation of this observation was that titin binds to actin in actively stretched muscle, thereby losing its ability to elongate its proximal region, shortening its free spring element, thereby increasing its stiffness and force upon stretch (Duvall et al., 2017; Herzog, 2018). Although these experiments, and associated interpretations, need further independent confirmation, they suggest that titin elasticity changes when a muscle is activated. The molecular details of this change in titin elasticity remain speculation, and even though we have tentatively proposed that it occurs through binding of titin to actin, this suggestion needs direct verification in live sarcomere or myofibril preparations, rather than fixed preparations or motility assays, as we believe that the arrangement of contractile, structural, and regulatory proteins that exists in sarcomeres is of crucial importance to the function and interactions of these proteins during muscle contraction.

### Advantages and Limitations of Myofibril Preparations for Studying Muscle Mechanics and Physiology

A great advantage of single myofibrils over any other muscle preparation is that myofibrils are comprised of sarcomeres that are strictly arranged in series. An in-series arrangement of sarcomeres means that each sarcomere and half-sarcomere must have the same internal force at each instant in time, independent of its material properties and regardless of its history of loading. The advantage of such an arrangement is that the force in each sarcomere can be measured with a force transducer at the end of the myofibril. Together with the relative ease of sarcomere length measurements in myofibrils, the entire mechanics of a sarcomere (force, length, and rate of change in length, that is its velocity of shortening or stretching) is known at any instant in time.

Another advantage of the myofibril preparation is that passive force arises essentially exclusively from the structural protein titin (Bartoo et al., 1997; Herzog et al., 2012). Therefore, the mechanical properties of a single, passive myofibril represent well the mechanical properties of the assembly of titin filaments in sarcomeres (Kellermayer et al., 1997). Furthermore, it is trivial in single myofibrils to manipulate cross-bridge binding, the amount of functional titin filaments, control the level of activation of each sarcomere, and manipulate the length and rate of change in length of sarcomeres accurately, thus presenting a plethora of possibilities for studying the role of titin under a variety of precisely known and precisely controllable contractile, mechanical, and activation conditions. We have used single myofibril and mechanically isolated sarcomere preparations to identify the changing force and stiffness properties of titin as a function of activation (calcium concentration), cross-bridge-associated force production, and contractile conditions, which allowed for the proposal of the three-filament (actin, myosin, and titin) theory of muscle contraction and force production (Leonard and Herzog, 2010; Herzog et al., 2015; Schappacher-Tilp et al., 2015; Herzog, 2018).

However, like any scientific approach, the myofibril preparation also has disadvantages. Primary among them the fact that there is no commercial system for myofibril and single sarcomere testing. Also, myofibril preparations are fragile, and it typically takes months of training before one can expect reliable results. Furthermore, it takes great patience to obtain and set up myofibrils for multiple activation cycles, and experiments not accounting for the force loss caused by repeat myofibril activation have led to erroneous conclusions (e.g., Cornachione et al., 2016), for example, in the proposed role of titin in cardiac muscle (Herzog, 2016). Other limitations of myofibril systems include that forces are often measured using relatively compliant nano-levers, thus there is some shortening of myofibrils and sarcomeres when force increases. For example, for our active force measurements in myofibrils, we use a nano-lever with a stiffness of 200 pN/nm. For a maximal isometric contraction of a myofibril at optimal sarcomere length, forces reach values of about 150 nN, thus the lever will bend by an amount of about 750 nm, causing the myofibril to shorten by the same amount. For an average myofibril comprised of 10 serially aligned sarcomeres at optimal length (10 × 2.4 µm for the rabbit psoas muscle), the total length before activation would be about 24 µm or 24,000 nm. A shortening of 750 nm due to lever compliance therefore constitutes a shortening of about 3% of the myofibril and thus the average sarcomere length. Or in other words, a sarcomere of 2.400 µm before activation would shorten to about 2.325 µm, a difference that would be hard to detect reliably with light microscopy. In order to minimize shortening during force production and maintain a good resolution for force measurements, we (and others) developed systems based on the principle of atomic force microscopy with negligible bending of the force lever (stiffness of 2–4 nN/nm) upon myofibril activation (e.g., Telley et al., 2006; Duvall et al., 2017).

Another limitation of the myofibril system is the accuracy with which absolute forces can be determined. Absolute force depends on the stiffness of the nano-force levers, the fixation technique (with or without glue, where on the lever the myofibril is attached), and the optical resolution of light microscopy. However, even though absolute forces might not be as accurate as one might wish, relative changes in force can be identified easily. For example, in experiments aimed at comparing force levels between an isometric reference contraction and a force enhanced state, forces are normalized to the isometric reference contraction, and force enhancement is provided as a percent change from the reference force. Therefore, any systematic error, for example, an error in nano-lever stiffness, would not affect this result. However, if one is interested in accurate absolute forces of a myofibril, factors that may affect the stiffness of the nano-levers used for force measurements, would need to be carefully accounted for.

When testing single myofibrils, sarcomere lengths are obtained using light microscopy which limits the spatial resolution to about 200 nm. For typical experiments, this resolution limit is not a problem. However, when trying to identify potential step-wise shortening of sarcomeres in the nano-metre range (e.g., Pollack et al., 1977; Pollack, 1986) or relate sub-nano-metre non-uniformities in half-sarcomere lengths to residual force enhancement properties (e.g., Rassier, 2012; Rassier and Pavlov, 2012), methods other than light microscopy need to be used. Optical resolution providing sub-nm accuracy are possible using electron microscopy or low angle x-ray diffraction (Ma and Irving, 2019). However, electron microscopy requires fixed specimens and cannot be performed on live muscles/myofibrils that are contracting and changing length, and while x-ray diffraction can be used with live and contracting muscle preparations, optimal diffraction patterns require specimens that are greater than single myofibrils/sarcomeres. Careful analysis of the capability of sarcomere length measurements in myofibril preparations using light microscopy suggests that a feasible accuracy may be in the 100 nm range, under extremely favourable conditions, changes of 50 nm might be detectable (Schmidt et al., 2021).

## Conclusion

Single myofibril and single sarcomere preparations offer great advantages to study the molecular mechanisms of muscle contraction with the sarcomeric proteins in their natural configuration. We believe that the proper protein configuration is of utmost importance when trying to determine the molecular details of muscle contraction, and differences in the structural arrangement of the contractile, regulatory and structural proteins might explain differences in observations made with myofibril, single molecule (e.g., Finer et al., 1994), or motility assay preparations (e.g., Riveline et al., 1998). Single myofibrils also have the advantage of serially arranged sarcomeres and the possibility to measure force, length, and rate of change in length for each sarcomere. However, myofibril experiments are technically difficult, require intensive and time-consuming training, and myofibrils are fragile, thus usually only allowing for a handful of experiments per specimen. Furthermore, the limits of accuracy in sarcomere lengths and force measurements, as well as the mechanics of serially linked mechanical systems must be kept in mind when interpreting results and formulating conclusions.

## References

[B1] Abbott B. C. Aubert X. M. (1952). The Force Exerted by Active Striated Muscle during and after Change of Length. J.Physiol. 117, 77–86. 10.1113/jphysiol.1952.sp004755 14946730 PMC1392571

[B2] Bartoo M. L. Linke W. A. Pollack G. H. (1997). Basis of Passive Tension and Stiffness in Isolated Rabbit Myofibrils. Am. J. Physiology-Cell Physiol. 273, C266–C276. 10.1152/ajpcell.1997.273.1.c266 9252465

[B3] Colomo F. Piroddi N. Poggesi C. te Kronnie G. Tesi C. (1997). Active and Passive Forces of Isolated Myofibrils from Cardiac and Fast Skeletal Muscle of the Frog. J. Physiol. 500 ( Pt 2) (Pt 2), 535–548. 10.1113/jphysiol.1997.sp022039 9147336 PMC1159402

[B4] Cornachione A. S. Leite F. Bagni M. A. Rassier D. E. (2016). The Increase in Non-cross-bridge Forces after Stretch of Activated Striated Muscle Is Related to Titin Isoforms. Am. J. Physiology-Cell Physiol. 310, C19–C26. 10.1152/ajpcell.00156.2015 PMC4698450 26405100

[B5] Dos Remedios C. Gilmour D. (2017). An Historical Perspective of the Discovery of Titin Filaments. Biophys. Rev. 9, 179–188. 10.1007/s12551-017-0269-3 28656582 PMC5498331

[B6] Duvall M. M. Jinha A. Schappacher-Tilp G. Leonard T. R. Herzog W. (2017). Differences in Titin Segmental Elongation between Passive and Active Stretch in Skeletal Muscle. J. Exp. Biol. 220, 4418–4425. 10.1242/jeb.160762 28970245

[B7] Duvall M. M. Gifford J. L. Amrein M. Herzog W. (2013). Altered Mechanical Properties of Titin Immunoglobulin Domain 27 in the Presence of Calcium. Eur. Biophys. J. 42, 301–307. 10.1007/s00249-012-0875-8 23224300

[B8] Edman K. A. Elzinga G. Noble M. I. (1982). Residual Force Enhancement after Stretch of Contracting Frog Single Muscle Fibers. J.Gen.Physiol. 80, 769–784. 10.1085/jgp.80.5.769 6983564 PMC2228643

[B9] Epstein M. Wong M. Herzog W. (2006). Should Tendon and Aponeurosis Be Considered in Series? J. Biomech. 39, 2020–2025. 10.1016/j.jbiomech.2005.06.011 16085074

[B10] Finer J. T. Simmons R. M. Spudich J. A. (1994). Single Myosin Molecule Mechanics: Piconewton Forces and Nanometre Steps. Nature 368, 113–119. 10.1038/368113a0 8139653

[B11] Freiburg A. Trombitas K. Hell W. Cazorla O. Fougerousse F. Centner T. (2000). Series of Exon-Skipping Events in the Elastic spring Region of Titin as the Structural Basis for Myofibrillar Elastic Diversity. Circ. Res. 86, 1114–1121. 10.1161/01.res.86.11.1114 10850961

[B12] Granzier H. Labeit S. (2002). Cardiac Titin: an Adjustable Multi‐functional spring. J. Physiol. 541, 335–342. 10.1113/jphysiol.2001.014381 12042342 PMC2290327

[B13] Helmes M. Trombitás K. Centner T. Kellermayer M. Labeit S. Linke W. A. (1999). Mechanically Driven Contour-Length Adjustment in Rat Cardiac Titin's Unique N2B Sequence. Circ. Res. 84, 1339–1352. 10.1161/01.res.84.11.1339 10364572

[B14] Herzog J. A. Leonard T. R. Jinha A. Herzog W. (2012). Are Titin Properties Reflected in Single Myofibrils? J. Biomech. 45, 1893–1899. 10.1016/j.jbiomech.2012.05.021 22677335

[B15] Herzog W. (2018). The Multiple Roles of Titin in Muscle Contraction and Force Production. Biophys. Rev. 10, 1187–1199. 10.1007/s12551-017-0395-y 29353351 PMC6082311

[B16] Herzog W. Leonard T. R. (2002). Force Enhancement Following Stretching of Skeletal Muscle. J.Exp.Biol. 205, 1275–1283. 10.1242/jeb.205.9.1275 11948204

[B17] Herzog W. (2016). Letter to the editor: Comments on Cornachione et al. (2016): "The increase in non-cross-bridge forces after stretch of activated striated muscle is related to titin isoforms"The increase in non-cross-bridge forces after stretch of activated striated muscle is related to titin isoforms. Am. J. Physiology-Cell Physiol. 311, C158–C159. 10.1152/ajpcell.00373.2015 PMC4967139 27413180

[B18] Herzog W. Powers K. Johnston K. Duvall M. (2015). A New Paradigm for Muscle Contraction. Front. Physiol. 6, 174–185. 10.3389/fphys.2015.00174 26113821 PMC4461830

[B19] Herzog W. Schappacher G. Duvall M. Leonard T. R. Herzog J. A. (2016). Residual Force Enhancement Following Eccentric Contractions: a New Mechanism Involving Titin. Physiology 31, 300–312. 10.1152/physiol.00049.2014 27252165

[B20] Hill A. V. (1953). The Mechanics of Active Muscle. Proc. R.Soc.Lond. 141, 104–117. 13047276 10.1098/rspb.1953.0027

[B21] Horowits R. Kempner E. S. Bisher M. E. Podolsky R. J. (1986). A Physiological Role for Titin and Nebulin in Skeletal Muscle. Nature 323, 160–164. 10.1038/323160a0 3755803

[B22] Horowits R. Maruyama K. Podolsky R. J. (1989). Elastic Behavior of Connectin Filaments during Thick Filament Movement in Activated Skeletal Muscle. J.Cell Biol. 109, 2169–2176. 10.1083/jcb.109.5.2169 2808523 PMC2115863

[B23] Horowits R. (1992). Passive Force Generation and Titin Isoforms in Mammalian Skeletal Muscle. Biophysical J. 61, 392–398. 10.1016/s0006-3495(92)81845-3 PMC1260255 1547327

[B24] Horowits R. Podolsky R. J. (1987). The Positional Stability of Thick Filaments in Activated Skeletal Muscle Depends on Sarcomere Length: Evidence for the Role of Titin Filaments. J.Cell Biol. 105, 2217–2223. 10.1083/jcb.105.5.2217 3680378 PMC2114850

[B25] Huxley A. F. (1957). Muscle Structure and Theories of Contraction. Prog. Biophys. Biophysical Chem. 7, 255–318. 10.1016/s0096-4174(18)30128-8 13485191

[B26] Huxley A. F. Niedergerke R. (1954). Structural Changes in Muscle during Contraction: Interference Microscopy of Living Muscle Fibres. Nature 173, 971–973. 10.1038/173971a0 13165697

[B27] Huxley A. F. Peachey L. D. (1961). The Maximum Length for Contraction in Vertebrate Striated Muscle. J.Physiol. 156, 150–165. 10.1113/jphysiol.1961.sp006665 13717107 PMC1359941

[B28] Huxley A. F. (1980). Reflections on Muscle. Liverpool: Liverpool University Press.

[B29] Huxley H. E. (2004). Fifty Years of Muscle and the Sliding Filament Hypothesis. Eur. J. Biochem. 271, 1403–1415. 10.1111/j.1432-1033.2004.04044.x 15066167

[B30] Huxley H. Hanson J. (1954). Changes in the Cross-Striations of Muscle during Contraction and Stretch and Their Structural Interpretation. Nature 173, 973–976. 10.1038/173973a0 13165698

[B31] Johnston K. Jinha A. Herzog W. (2016). The Role of Sarcomere Length Non-uniformities in Residual Force Enhancement of Skeletal Muscle Myofibrils. R. Soc. Open Sci. 3, 150657. 10.1098/rsos.150657 27069655 PMC4821266

[B32] Johnston K. Moo E. K. Jinha A. Herzog W. (2019). On Sarcomere Length Stability during Isometric and post-active-stretch Isometric Contractions. J.Exp.Biol. 222, 209924. 10.1242/jeb.209924 31704896

[B33] Joumaa V. Rassier D. E. Leonard T. R. Herzog W. (2008). The Origin of Passive Force Enhancement in Skeletal Muscle. Am. J. Physiology-Cell Physiol. 294, C74–C78. 10.1152/ajpcell.00218.2007 17928540

[B34] Kellermayer M. S. Z. Smith S. B. Granzier H. L. Bustamante C. (1997). Folding-unfolding Transitions in Single Titin Molecules Characterized with Laser Tweezers. Science 276, 1112–1116. 10.1126/science.276.5315.1112 9148805

[B35] Labeit D. Watanabe K. Witt C. Fujita H. Wu Y. Lahmers S. (2003). Calcium-dependent Molecular spring Elements in the Giant Protein Titin. Proc. Natl. Acad. Sci. U.S.A. 100, 13716–13721. 10.1073/pnas.2235652100 14593205 PMC263879

[B36] Leonard T. R. Duvall M. Herzog W. (2010). Force Enhancement Following Stretch in a Single Sarcomere. Am. J. Physiology-Cell Physiol. 299 (6), C1398–C1401. 10.1152/ajpcell.00222.2010 20844251

[B37] Leonard T. R. Herzog W. (2010). Regulation of Muscle Force in the Absence of Actin-Myosin-Based Cross-Bridge Interaction. Am. J. Physiology-Cell Physiol. 299, C14–C20. 10.1152/ajpcell.00049.2010 20357181

[B38] Linke W. A. Popov V. I. Pollack G. H. (1994). Passive and Active Tension in Single Cardiac Myofibrils. Biophysical J. 67, 782–792. 10.1016/s0006-3495(94)80538-7 PMC1225421 7948691

[B39] Linke W. A. Rudy D. E. Centner T. Gautel M. Witt C. Labeit S. (1999). I-band Titin in Cardiac Muscle Is a Three-Element Molecular spring and Is Critical for Maintaining Thin Filament Structure. J.Cell Biol. 146, 631–644. 10.1083/jcb.146.3.631 10444071 PMC2150553

[B40] Linke W. A. (2018). Titin Gene and Protein Functions in Passive and Active Muscle. Annu. Rev. Physiol. 80, 389–411. 10.1146/annurev-physiol-021317-121234 29131758

[B41] Llewellyn M. E. Barretto R. P. J. Delp S. L. Schnitzer M. J. (2008). Minimally Invasive High-Speed Imaging of Sarcomere Contractile Dynamics in Mice and Humans. Nature 454, 784–788. 10.1038/nature07104 18600262 PMC2826360

[B42] Ma W. Irving T. C. (2019). X-ray Diffraction of Intact Murine Skeletal Muscle as a Tool for Studying the Structural Basis of Muscle Disease. JoVE (Journal of Visualized Experiments) 149, e59559. 10.3791/59559 PMC6765332 31380854

[B43] Maruyama K. (1995). Birth of the Sliding Filament Concept in Muscle Contraction. J. Biochem. 117, 1–6. 10.1093/oxfordjournals.jbchem.a124692 7775372

[B44] Mendoza A. C. Rassier D. E. (2020). Extraction of Thick Filaments in Individual Sarcomeres Affects Force Production by Single Myofibrils. Biophysical J. 118, 1921–1929. 10.1016/j.bpj.2020.03.007 PMC7176582 32251620

[B45] Moo E. K. Fortuna R. Sibole S. C. Abusara Z. Herzog W. (2016). *In Vivo* sarcomere Lengths and Sarcomere Elongations Are Not Uniform across an Intact Muscle. Front. Physiol. 7, 187–189. 10.3389/fphys.2016.00187 27252660 PMC4879144

[B46] Morgan D. (1994). An Explanation for Residual Increased Tension in Striated Muscle after Stretch during Contraction. Exp. Physiol. 79, 831–838. 10.1113/expphysiol.1994.sp003811 7818869

[B47] Peterson D. R. Rassier D. E. Herzog W. (2004). Force Enhancement in Single Skeletal Muscle Fibres on the Ascending Limb of the Force-Length Relationship. J.Exp.Biol. 207, 2787–2791. 10.1242/jeb.01095 15235007

[B48] Pollack G. H. Iwazumi T. Ter Keurs H. E. D. J. Shibata E. F. (1977). Sarcomere Shortening in Striated Muscle Occurs in Stepwise Fashion. Nature 268, 757–759. 10.1038/268757a0 895878

[B49] Pollack G. H. (1986). Quantal Mechanisms in Cardiac Contraction. Circ. Res. 59, 1–8. 10.1161/01.res.59.1.1 3731408

[B50] Rassier D. E. Herzog W. Wakeling J. Syme D. A. (2003). Stretch-induced, Steady-State Force Enhancement in Single Skeletal Muscle Fibers Exceeds the Isometric Force at Optimum Fiber Length. J. Biomech. 36, 1309–1316. 10.1016/s0021-9290(03)00155-6 12893039

[B51] Rassier D. E. Pavlov I. (2012). Force Produced by Isolated Sarcomeres and Half-Sarcomeres after an Imposed Stretch. Am. J. Physiology-Cell Physiol. 302, C240–C248. 10.1152/ajpcell.00208.2011 21998143

[B52] Rassier D. E. (2012). Residual Force Enhancement in Skeletal Muscles: One Sarcomere after the Other. J. Muscle Res. Cel Motil 33, 155–165. 10.1007/s10974-012-9308-7 22729612

[B53] Riveline D. Ott A. Jülicher F. Winkelmann D. A. Cardoso O. Lacapère J.-J. (1998). Acting on Actin: the Electric Motility Assay. Eur. Biophys. J. 27, 403–408. 10.1007/s002490050147 9691469

[B54] Schappacher-Tilp G. Leonard T. Desch G. Herzog W. (2015). A Novel Three-Filament Model of Force Generation in Eccentric Contraction of Skeletal Muscles. PLoS ONE 10, e0117634. 10.1371/journal.pone.0117634 25816319 PMC4376863

[B55] Schappacher-Tilp G. (2018). Titin-mediated Thick Filament Activation Stabilizes Myofibrils on the Descending Limb of Their Force-Length Relationship. J. Sport Health Sci. 7, 326–332. 10.1016/j.jshs.2018.05.002 30356636 PMC6189248

[B56] Schmidt J. Jinha A. Herzog W. (2021). Sarcomere Length Measurement Reliability in Single Myofibrils. J. Biomech. 126, 110628. 10.1016/j.jbiomech.2021.110628 34274869

[B57] Telley I. A. Denoth J. Stüssi E. Pfitzer G. Stehle R. (2006). Half-sarcomere Dynamics in Myofibrils during Activation and Relaxation Studied by Tracking Fluorescent Markers. Biophysical J. 90, 514–530. 10.1529/biophysj.105.070334 PMC1367057 16239326

[B58] Trombitas K. Granzier H. (1997). Actin Removal from Cardiac Myocytes Shows that Near Z Line Titin Attaches to Actin while under Tension. Am. J. Physiology-Cell Physiol. 273, C662–C670. 10.1152/ajpcell.1997.273.2.c662 9277364

